# A Randomised Controlled Trial Testing the Efficacy of an Educational Website About Functional Abdominal Pain for Children and Adolescents

**DOI:** 10.1002/ejp.70249

**Published:** 2026-03-18

**Authors:** Verena Neß, Clarissa Humberg, Lisa‐Marie Rau, Leandra Eidt, Thomas Berger, Martin Claßen, Nils Christian Syring, Jens Berrang, Christine Vietor, Stephan Buderus, Gerrit Hirschfeld, Christina Becker‐Emden, Julia Wager

**Affiliations:** ^1^ German Paediatric Pain Centre, Children's and Adolescents' Hospital Datteln Datteln Germany; ^2^ Department of Children's Pain Therapy and Paediatric Palliative Care Witten/Herdecke University, Faculty of Health, School of Medicine Witten Germany; ^3^ PedScience Research Institute Datteln Germany; ^4^ Hospital Group Gesundheit Nord, Klinikum Bremen Mitte, Centre for Children and Parents Prof. Hess, Paediatric Clinic Bremen Germany; ^5^ Hospital Dortmund, Witten/Herdecke University, Faculty of Health, School of Medicine, Witten Dortmund Germany; ^6^ Techniker Krankenkasse Hamburg Germany; ^7^ GFO Clinics Bonn, St. Marienhospital Bonn Bonn Germany; ^8^ Hochschule Bielefeld HSBI Bielefeld Germany

**Keywords:** functional abdominal pain, multilevel analysis, paediatrics, patient portals, randomised controlled trial

## Abstract

**Background:**

Functional abdominal pain (FAP) is a severely debilitating condition affecting approximately 11.7% of children and adolescents worldwide, often persisting into adulthood and significantly restricting daily life. Health literacy is essential for treatment success. To improve knowledge about the biopsychosocial factors influencing FAP and support its management, we developed an educational multimedia website (https://meine‐bauchstelle.com).

**Methods:**

In a multicenter randomised controlled trial (RCT), patients with FAP (*N* = 166, age 5–17 years, *M* = 10.8, SD = 3.31; 53.6% female) and their parents were randomly assigned to an intervention group (IG: access to the website before the first measurement) or a control group (CG: no access to the website during data collection). The primary outcome was health literacy (knowledge and health behaviour) and secondary outcomes were abdominal pain symptoms and the physician‐patient/parent interactions. Group differences were analysed using *t*‐tests and multilevel models.

**Results:**

Patients in the IG who visited the website demonstrated significantly higher initial knowledge scores compared to the CG. Moreover, they reported significantly less pain‐related disability across three assessments over 3 months. Parents who visited the website rated the physician‐parent interaction significantly more positively than those in the CG.

**Conclusions:**

The educational website efficiently increased patient knowledge about FAP and improved certain pain‐related behaviours. It serves as an effective tool in conveying information about FAP in clinical practice. Future applications could extend to preventive measures in schools and other settings.

**Significance Statement:**

The educational website about functional abdominal pain is effective in transferring knowledge, reducing pain‐related disability, and improving physician‐parent interactions. It can support physicians in day‐to‐day clinical practice by referring patients to the website for further information and might be extended as a preventive measure in schools.

## 1 Introduction

Functional abdominal pain (FAP) is a psychosomatic condition affecting the gut‐brain interaction (Mayer et al. 2015), characterised by changes in bi‐directional communication (Mukhtar et al. 2019). FAP is classified according to ROME IV diagnostic criteria (Hyams et al. 2016), which define FAP as episodic or continuous abdominal pain occurring 4+ times per month for longer than 2 months. FAP is diagnosed when detectable somatic conditions have been ruled out (Korterink et al. 2015). There are four subtypes of functional abdominal pain disorders (FAPDs): functional dyspepsia, abdominal migraine, irritable bowel syndrome, and FAP not otherwise specified. Its development and persistence are influenced by a complex interplay of biopsychosocial factors (Wager and Zernikow 2014).

Worldwide, FAP affects approximately 11.7% of children and adolescents (Koen Vermeijden et al. 2025). These numbers are alarming, given that FAP restricts daily functioning, including school absenteeism and health‐related quality of life (Assa et al. 2015; Varni et al. 2015). Around 35% of children with FAP are affected into adulthood, with about 48% developing chronic pain in other body regions (Walker et al. 2010). Given these impairments and comorbidities, effective treatment and prevention of FAP is of utmost importance.

Despite its prevalence, families often reject FAP as a diagnosis and many seek additional clinical investigations (Claßen 2018; Crushell et al. 2003). This reaction is often accompanied by solicitous parental behaviours, which may inadvertently exacerbate FAP symptoms (Jolliffe and Nicholas 2004). Some parents discourage their child from active coping (Dunn‐Geier et al. 1986), which is important for symptom improvement (Högström et al. 2022).

To increase acceptance of FAP diagnoses, both parents and patients need strong health literacy (Kisling et al. 2021; Louw et al. 2016). However, physicians have limited time to elucidate the condition (Østbye et al. 2005). When patients and parents possess prior knowledge, they can better integrate new information from physicians into their cognitive frameworks (Zheng et al. 2024). This knowledge is essential for adopting functional behaviours (Louw et al. 2020; Nordin et al. 2017). Multimedia learning is an effective educational tool for children and adolescents, but its success may vary by age and sex (Kisling et al. 2021).

Our study aimed to provide a multimedia platform about FAP and its management, accessible to children, adolescents, and their parents. This setup is closely related to pain neuroscience education approaches (PNE), which emphasises communication and information transfer in chronic pain treatment to modify cognition about one's pain experience and achieve clinical benefits (Louw and Riera‐Gilley 2024). In a randomised controlled trial (RCT), patient‐parent dyads were assigned to either an intervention group (IG), which received website access, or a control group (CG), which had no access. The primary outcome was health literacy (including knowledge and health behaviours), while secondary outcomes included pain symptoms and physician‐patient/physician‐parent interactions. We expected that children in the IG would demonstrate better health literacy, pain outcomes, and physician‐patient interactions compared to the CG. Similarly, that parents in the IG would show better health literacy and physician‐parent interactions. Exploratory analyses examine differences by age and sex.

## 2 Methods

### 2.1 Website Design

A website about functional abdominal pain (FAP) for children, adolescents, and their parents—funded by the Innovation Committee of the Federal Joint Committee—was the cornerstone of the project, “Knowledge empowers! Empowerment of parent and child with functional abdominal pain”. Website content was developed by a multidisciplinary team of researchers, paediatric gastroenterologists, health insurance staff, and children/adolescents affected by FAP as well as their parents. The development process involved several preparatory steps. First, interviews were conducted with child–parent dyads (*N* = 6) and paediatric gastroenterologists (*N* = 11) to identify key topics for inclusion on the website, starting in November 2020. A subsequent systematic literature search and additional internet research were conducted to extract relevant information and assess existing resources. Relevant material was prepared, and feedback on its comprehensibility, quality, and clarity was sought from children and adolescents with FAP. After the website was refined accordingly, usability tests were carried out. Children and adolescents with FAP (*N* = 7) and their parents (*N* = 6) freely navigated the website under the observation of instructors (G.H., V.N.), who tracked navigation behaviour. Questions from the instructors guided the assessment of whether key information could be found easily on the website and to identify potential navigation difficulties. Following the usability tests, the children and parents completed questionnaires to provide additional feedback. Evaluation of the usability tests revealed positive feedback regarding website content exceeding pre‐established benchmarks (Moshagen and Thielsch 2010; Thielsch and Hirschfeld 2019; Meinald T. Thielsch and Salaschek 2020) (see Table 1). Further refinements were made to improve usability and address any technical difficulties identified during testing. Website development was completed in May 2022.

**TABLE 1:** Results of the usability tests for children/adolescents and their parents.

Scale	Children	Parents	Benchmarks
Usability	5.76 (0.96)	5.33 (0.87)	4.73 (1.43)^a^
Content	6.64 (0.45)	6.29 (0.90)	5.06 (0.33)^b^
Aesthetics	6.34 (0.83)	6.07 (1.30)	4.60 (1.37)^c^

*Note:* Mean values (SD) are displayed. Evaluation for all scales ranges from 1 to 7: 1 = “*do not agree at all*”, 2 = “*do not agree*”, 3 = “*somewhat disagree*”, 4 = “*neutral*”, 5 = “*tend to agree*”, 6 = “*agree*”, 7 = “*fully agree*”.

^a^ (Thielsch and Salaschek 2020).

^b^ (Thielsch and Hirschfeld 2019).

^c^ (Moshagen and Thielsch 2010).

The website is designed to appeal to children and adolescents, and therefore features a construction site theme that aligns with its name, “meine Bau(ch)stelle” (https://meine‐bauchstelle.com)—a play on words, incorporating the words “stomach” and “construction site”. The website was designed to be entertaining and engaging, containing numerous graphics, videos, and other multimedia elements. A gender‐neutral main character, Sam, guides the visitor through the website and is the main protagonist of the videos and most graphics. The main sections of the website cover (1) general knowledge about the gastrointestinal tract, (2) abdominal pain, (3) functional abdominal pain, and (4) effective management of functional abdominal pain. To cater to different audiences, the website offers three tailored entry points: one for children (ages 5–10 years), one for adolescents (≥ 11 years), and one for parents. While most content overlaps across these groups (only (1) is left out of the parent version), the design and presentation are tailored to each group. For the younger age group sufficient reading competency cannot be expected, therefore the children's area is equipped with sound buttons for each text block, allowing younger users or those less confident in reading to listen to the website content. A key feature of the website is an educational video about FAP, which explains its development, contributing factors, subtypes, and management strategies, summarising its key concepts in an accessible and engaging way. Website content is in accordance with criteria of the guidelines for evidence‐based health information developed by the German Network for Evidence‐based Medicine in cooperation with the health sciences department at the University of Hamburg (Lühnen et al. 2015).

### 2.2 Study Design and Procedure

To test the efficacy of the educational website, a multicenter randomised controlled trial (RCT) was conducted. Children and adolescents suspected of presenting with FAP, along with their parents, were randomised into either an intervention group (treatment as usual plus access to the website) or a control group (treatment as usual without access to the website during data collection). Data were collected at three measurements: immediately following the initial physician's appointment (T_1_), and 1 month (T_2_) and 3 months (T_3_) after the physician's appointment. The two groups were compared across all outcome variables.

### 2.3 Randomization

To conduct the RCT, participants were randomly assigned to either an intervention group (IG) or control group (CG). Only children with suspected FAP were eligible. The IG received access to the website *before* their first visit to the clinic. To accomplish this, study nurses monitored upcoming appointments scheduled within the next 2–3 weeks for referrals due to symptoms likely leading to an FAP diagnosis. The eligible diagnoses for randomization were predefined by the participating paediatric gastroenterologists (T.B., M.C., J.B., S.B.). At least one of the following diagnoses had to be mentioned: irritable bowel syndrome (IBS), functional dyspepsia, abdominal migraine, functional abdominal pain not otherwise specified, constipation with abdominal pain, fructose malabsorption, or lactose malabsorption. Although fructose and lactose malabsorption are not FAP diagnoses, in clinical practice, children with these tentative diagnoses are often later diagnosed with FAP. For randomization, children who fulfilled all inclusion criteria (see *Recruitment*) were listed in a table with group assignments provided by a biometrician (G.H.).

### 2.4 Recruitment

Recruitment took place in three paediatric gastroenterology departments in Germany (Centre for Children and Parents Prof. Hess, Paediatric Clinic, Bremen, Children's and Adolescents' Hospital Datteln, and Hospital Dortmund) between June 2022 and December 2023. The study design had a unique feature: we aimed to assess the website's impact when presented to participants *before* the first appointment, particularly regarding the physician‐patient interaction. At the same time, experiences from prior studies taught us that recruitment success is higher when families are recruited on‐site. Therefore, group randomization took place before recruitment, and the intervention (access to the website) started before the first measurement point (see *Randomization*). As a result, no traditional baseline measurement was conducted, since the intervention had already commenced before the first measurement. The control group received access to the website after data collection was complete.

All children with an appointment at one of the three paediatric gastroenterology departments were screened for eligibility. Inclusion criteria were children and adolescents between 5 and 17 years of age, suspected FAP, and no presentation to one of the participating sites for abdominal pain in the prior 12 months. Exclusion criteria were no FAP diagnosis and insufficient proficiency in the German language. The accompanying parent was also eligible for the study. Children suspected of having a FAP diagnosis were randomly assigned to either the control group (CG) or intervention group (IG) (see *Randomization*). To maximise awareness and usage of the website before their appointment, families in the IG were contacted through various channels. They were called by study nurses to inform them about the website (without mentioning the study). Additionally, an e‐mail including the website link and a postcard promoting the website was sent to families.

The initial physician's visit proceeded as usual for all eligible families, with physicians blinded to the patients' and parents' group assignments. If a FAP diagnosis was confirmed during the appointment and the family had sufficient proficiency in German, they were informed about the study and invited to participate. Informed assent and consent forms were signed. For families previously randomised to the IG, they were handed over a second postcard reminding them to use the website. All participants (both child and parent) were asked to complete assessments at three time points. The first questionnaire was completed on tablets directly after recruitment, following the initial physician's appointment (T_1_). Follow‐up assessments (T_2_ and T_3_) were assessed by an online questionnaire. To maximise participation in the follow‐up measurements, reminder e‐mails were sent 5 days (Reminder 1) and 8 days (Reminder 2) after the initial invitation if questionnaires were not yet completed. If participants did not respond to either e‐mail, study nurses contacted participants via phone 7 days after Reminder 2. The last questionnaires of the final assessment (T_3_) were acquired in February 2024. Completion of the study was achieved in July 2024.

### 2.5 Ethics

Ethics approval for the project was granted by the committee of Witten/Herdecke University (reference number 185/2020). Additional endorsements were obtained from the medical chamber of Bremen (application number 743), the Ethics Committee of the Medical Association of Westphalia‐Lippe and Westfälische Wilhelms University of Münster (file number 2020‐852‐b‐S), and the Medical Association of North Rhine (serial number: 2021140). The study was previously registered at the German Clinical Trials Register (ID: DRKS00028799) on June 20, 2022.

### 2.6 Measurements and Variables

Three versions of the questionnaire were provided, one for young children (5–7 years), one for children and adolescents (8–17 years), and one for parents. Parents were responsible for entering the responses of young children on the tablet, as young children typically have limited reading and writing abilities. However, parents were encouraged to actively involve their children by reading the questions to them and asking them for their answers. For children aged 8 years and older, parents were allowed to help their children read the questions and instructions, if necessary, but were instructed not to influence their child's responses. Each questionnaire included demographic questions to assess variables such as sex, age, and migration.

#### 2.6.1 Primary Outcomes Children and Adolescents

The primary outcome of this study is health literacy, which comprises knowledge and health behaviour.

Abdominal Pain Knowledge was assessed for children 8 years and older using the child version of the Abdominal Pain Knowledge Questionnaire (A‐PKQ) (Neß et al. 2024). This measure can be used for children 8 years and older. It comprises 12 single‐choice items suitable for our study population (the published version comprises 10 items) which were coded as binary (0 = “*wrong*”, 1 = “*correct*”; range: 0–12). The child version of the A‐PKQ includes general knowledge questions about the gastrointestinal tract, abdominal pain, functional abdominal pain, and abdominal pain management (e.g., “Which statement about stool shape and consistency is correct?”). These categories correspond to the domains of our developed website.

To measure health behaviour in the context of abdominal pain, three different measures were used.

The Paediatric Pain Coping Inventory—revised (PPCI‐r) assesses handling of pain (Hechler et al. 2008). The inventory is introduced with the statement, “When I'm in pain or something hurts…”, followed by the various options for dealing with pain (e.g., “… I ask for medication.”). The PPCI‐r is answered on a 3‐point scale (1 = “*almost never*”, 2 = “*sometimes*”, 3 = “*often*”) and includes three subscales. Two subscales assess behavioural coping strategies: “Passive pain coping” (10 items; range: 10–30; e.g., “When I'm in pain or something hurts… I go to bed.”) and “Search for social support” (8 items; range: 8–24 e.g., “When I'm in pain or something hurts… I want to be held in someone's arm.”). The other subscale assesses cognitive coping strategies: “Positive self instruction” (7 items; range: 7–21 e.g., “When I'm in pain or something hurts… I try not to think about the pain.”).

Missed school days in the last 4 weeks due to abdominal pain were assessed by the question: “Please indicate the number of days you were unable to go to school due to abdominal pain“. Participants were also asked about partial school days missed: “Please indicate the number of days you went home early or arrived late to school due to abdominal pain” (range: 0–20).

In addition, the Paediatric Pain Disability Index (PPDI) was used to assess impairments due to pain over the last 4 weeks. The question asks, “If you have pain, how often has it bothered you during the following activities in the last 4 weeks?” (Hübner et al. 2009). The PPDI comprises 12 items (e.g., “sports, homework, sleep”) answered on a 5‐point scale (1 = “*never*”, 2 = “*rarely*”, 3 = “*sometimes*”, 4 = “*frequently*”, 5 = “*always*”; range of sum score: 12–60).

#### 2.6.2 Secondary Outcomes Children and Adolescents

Abdominal pain symptoms were assessed in the child and adolescent questionnaire by the Abdominal Pain Index (API) (Laird et al. 2015). The API includes four items: frequency of abdominal pain (“In the past 2 weeks, how often have you had abdominal pain (stomach aches)?”; 0 = “*not at all*”, 1 = “*1 or 2 days*”, 2 = “*3 or 4 days*”, 3 = “*5 or 6 days*”, 4 = “*most days*”, 5 = “*every day*”), daily frequency of abdominal pain (0 = “*none”*, 1 = “*once a day*”, 2 = “*two or three times a day*”, 3 = “*four or five times a day*”, 4 = “*six or more times during the day*”, 5 = “*constant during the day*”), duration of abdominal pain (0 = “*no pain*”, 1 = “*a few minutes*”, 2 = “*about half an hour*”, 3 = “*about an hour*”, 4 = “*between 1 and 2 h*”, 5 = “*3 or 4 h*”, 6 = “*5 or 6 h*”, 7 = “*most of the day*”, 8 = “*all day (it never completely stops)*”), and intensity of abdominal pain (ten‐point scale: 0 = “*no pain*”, 10 = “*the most pain possible*”) in the past 2 weeks. A final composite score, converted to a five‐point scale ranging from 0 to 4, is calculated from these four items.

For physician‐patient interaction (assessed at T1 only), physicians completed an evaluation form (practitioner's evaluation). This questionnaire was used so that healthcare professionals could record how patients behaved during the consultation from the healthcare professional's perspective. They rated children and adolescents on their communication, understanding, reaction, and behaviour towards the physician's information (e.g., “The patient was able to follow the explanations well and understood them well.”). Ratings used a five‐point‐scale from 1 = *most positive* (e.g., “The patient has made specific and needs‐based inquiries”) to 5 = *most negative* (e.g., “The patient has made no or inappropriate inquiries”).

Moreover, patients aged 8 years and older answered the “Fragebogen zur Arzt‐Patienten‐Interaktion” (FAPI; English: Physician‐Patient Interaction Questionnaire) (Bieber et al. 2011). In order to take both perspectives into account, the patients' view on the behaviour of the doctors treating them were also recorded to measure the doctor‐patient interaction. Items were measured on a five‐point scale (1 = “does not apply” to 5 = “*exactly right*”; e.g., “The doctor understood and took my problems and concerns seriously.”). The final score was calculated as the mean value across all 14 items.

#### 2.6.3 Primary Outcomes Parents

For parents, health literacy was also the primary outcome.

To assess their knowledge, parents completed the parent version of the A‐PKQ. Like the child version, it comprises 12 single‐choice items suitable to the respective study population (the published version comprises 10 items; coded binary: 0 = “*wrong*”, 1 = “*correct*”; range: 0–12) about topics related to abdominal pain, functional abdominal pain, and abdominal pain management (including support and nutrition; e.g., “How does functional abdominal pain change when you pay special attention to your stomach?”). These categories correspond to the domains featured on the parent area of our website.

Parental behaviour regarding their child's pain was assessed using the “Inventar für schmerzbezogenes Elternverhalten” (ISEV‐E; English: “Pain‐related Parent Behavior Inventory”) (Frerker et al. 2018). The questionnaire begins with: “If my son/daughter is in pain,…”, and presents various coping behaviours (e.g., “… I am particularly kind to him”). Each item was rated on a five‐point scale (1 = “*never*”, 2 = “*rarely*”, 3 = “*sometimes*”, 4 = “*often*”, 5 = “*very often*”). The ISEV‐E includes three subscales: “Discouragement” (7 items; range: 7–35; e.g., “If my son/daughter is in pain… I become impatient.”), “Solicitousness” (6 items; range: 6–30; e.g., “If my son/daughter is in pain… I take special care of him/her.”), and “Distraction” (4 items; range: 4–20, e.g., “If my son/daughter is in pain… I encourage him/her to do something nice.”).

#### 2.6.4 Secondary Outcomes Parents

For the physician‐parent interaction (assessed at T1 only), physicians completed an evaluation form assessing their interaction with the parents (practitioner's evaluation). This questionnaire was used so that healthcare professionals could record how guardians behaved during the consultation from the healthcare professional's perspective. The same aspects were evaluated as for the children and adolescents, including communication, understanding, reaction, and behaviour towards the physician's information (e.g., “The legal guardian was able to follow the explanations well and understood them well.”). The evaluation used a five‐point‐scale, from 1 = *most positive* (e.g., “The custodian has made specific and needs‐based inquiries”) to 5 = *most negative* (e.g., “The custodian has made no or inappropriate inquiries”).

In addition, parents also completed the Questionnaire of Physician‐Patient Interaction (FAPI). Like for the patients, also the parents' view on the behaviour of the doctors treating their child were also recorded to measure the doctor‐parent interaction. Items were measured on a five‐point scale (1 = “does not apply” to 5 = “*exactly right*”; e.g., “The doctor understood and took my problems and concerns seriously.”), and the final score was calculated as the mean value across all 14 items.

Additionally, a global item was included in the parent assessment to evaluate their confidence managing their child's abdominal pain: “How confident do you feel in dealing with your child's pain”? This was measured on a ten‐point scale (0 = “*not confident at all*” to 10 = “*absolutely confident*”).

### 2.7 Questions Regarding the Website

To assess website engagement, participants in the intervention group (IG) were asked whether they visited the website, the duration of visits, and which sections they explored. Moreover, they provided feedback on the website, including a general evaluation, suggestions for improvement, and whether they found the content useful and easy to understand.

The control group (CG) was asked only a single question at the final measurement (T_3_) assessing whether they were aware of the website to control whether the website content had been compromised.

### 2.8 Hypotheses

The study design (see 2.2) and the reference time frame of the measures (see 2.3) had influenced the formulation of hypotheses for this trial.

Most child outcomes related to pain impairments referred to the last 2 to 4 weeks. The website link for the IG was sent, on average, 2 weeks before the physician's appointment. While it was expected that abdominal pain knowledge would be higher in the IG at the first assessment, it was assumed that this knowledge would not lead to immediate changes in health behaviour (e.g., pain coping, pain‐related disability, and missed school days) or abdominal pain symptoms. Therefore, no group differences between the IG and CG were expected at the first assessment (T_1_), and any lack of group differences would indicate successful randomization. It was, however, expected that physician‐patient interactions would improve in the IG compared to CG. Furthermore, it was hypothesized that children in the IG would demonstrate more favourable outcomes over time across all measured variables, resulting in significant interaction effects between time and group.

Parent questionnaires did not specify reference time frames, meaning that questions about behaviour were not tied to a specific time frame in the past. Therefore, group differences in all primary and secondary outcomes were expected from the first assessment, as parents in the IG may have already applied the knowledge acquired from the website. Moreover, interaction effects between time and group were expected over time.

### 2.9 Statistical Analysis

#### 2.9.1 Sample Size Calculation

For our analyses, a sample size of *n* = 96 (*n* = 48 per group) was required to detect a small time by group interaction (*f* = 0.15) with a power of 90% at a significance level of 5% (*α* = 0.05), assuming a correlation of *r* = 0.5 between measurements. Based on prior studies, we expected a 50% dropout rate at T_3_. It was therefore planned to recruit *N* = 192 patients, with *n* = 96 assigned to each of the intervention and control groups.

#### 2.9.2 Multiple Imputation

Missing data for sex and age variables were completed using recruitment lists. To handle all other missing values, multiple imputation was performed using the packages ‘*mice*’ (van Buuren and Groothuis‐Oudshoorn 2011) and ‘*mitml*’ (Grund et al., n.d.) in R and RStudio (Version 4.1.1; Posit team 2023b; R Core Team 2021). The predictor matrix included all demographic variables, group, and time variables as predictors, but these variables were set to zero as outcome variables. The class variable (id) was explicitly defined. Moreover, only variables correlating at least *r*_*τ*_ = 0.1 with the outcome variable were selected as predictors. The imputation method “2l.pan” was used for mixed effects models. In total, 100 imputations were run (seed = 10,092,024), generating 100 imputed datasets. Missing data in each set were replaced with the most plausible values predicted by the model. Each of the 100 imputed datasets was then analysed (see 2.5.4 Statistical Procedure).

#### 2.9.3 Analysis Plan

Two procedures were used to analyse the data. First, an Intention‐to‐treat (ITT) analysis was performed, followed by a Per‐protocol (PP) analysis. For the PP analysis, only participants who reported visiting the website at T_1_ and/or T_2_ were included in the IG (website visited). Those who were originally assigned to the IG but who visited the website only at T_3_ or not at all were reassigned to the CG (website not visited) for this analysis. Moreover, all CG participants who reported hearing about the website were excluded from the PP analysis.

#### 2.9.4 Statistical Procedure

Before conducting the main analyses, dropout patterns were analysed separately for children/adolescents and parents. Multiple group comparisons were corrected using the Benjamini‐Hochberg procedure. Participants who completed at least at the first measurement (T_1_) were categorised into two groups. The ‘dropout’ group comprised those who participated exclusively at T_1_. The ‘completer’ group comprised all those who participated at T_1_ and at least at one follow‐up assessment (T_2_ and/or T_3_). Comparisons between dropout and completer groups were done by testing for significant differences in all outcome variables, as well as patients' and parents' sex and age. *P*‐values and mean scores are reported in cases of significance. Moreover, differences in pain severity (API) and disability (PPD) were assessed at T1 by group comparisons with multiply imputed data between participants of the intervention group who visited the website and those who did not. Furthermore, it was tested whether age of participants in the intervention group, split into two groups corresponding website sections for children in primary and secondary school (< 11 years; ≥ 11 years), influenced whether the website was visited or not.

Differences between FAP subtypes were not analysed. Based on clinical experience and prior literature, most children are diagnosed with “FAP not otherwise specified” (Seetharaman et al. 2025). Given the targeted sample size, the small number of patients with other FAP subtypes would not only have limited statistical analyses and interpretation of results but also may have jeopardised data anonymity.

After multiple imputation, all child and parent outcomes at T_1_ were analysed using *t*‐tests or Mann–Whitney *U*‐tests. Data distribution was assessed via Shapiro–Wilk‐tests to determine whether to apply a parametric or non‐parametric procedure. Data across all measurements were analysed using separate multilevel linear models (MLMs) for each outcome, including an interaction term for group and time. Final results report pooled estimates and *p*‐values.

Additional sensitivity analyses were done for both participants and parents to investigate the potential influence of age and sex on the various outcomes. For this, age and sex were included as additional predictors in the multilevel linear models (MLM) to explore whether accounting for these variables explained more variance and increased statistical power. Furthermore, the development of each outcome over time was evaluated descriptively.

Dropout analyses, descriptive statistics, *t*‐tests, and MLM analyses were conducted using R and RStudio (Version 4.4.1; (Posit team 2023a; R Core Team 2021)). For dropout analyses, the *compareGroups* package was used (Subirana et al. 2014); for the MLM analyses and pooled *t*‐tests, the *lmerTest* (Kuznetsova et al. 2017) package was used. Mann–Whitney U‐tests and Shapiro–Wilk‐tests were calculated using the ‘*rstatix*’ package (Kassambara 2023).

## 3 Results

### 3.1 Demographics and Dropout Analysis

#### 3.1.1 Sample and Participation

**FIGURE 1:**
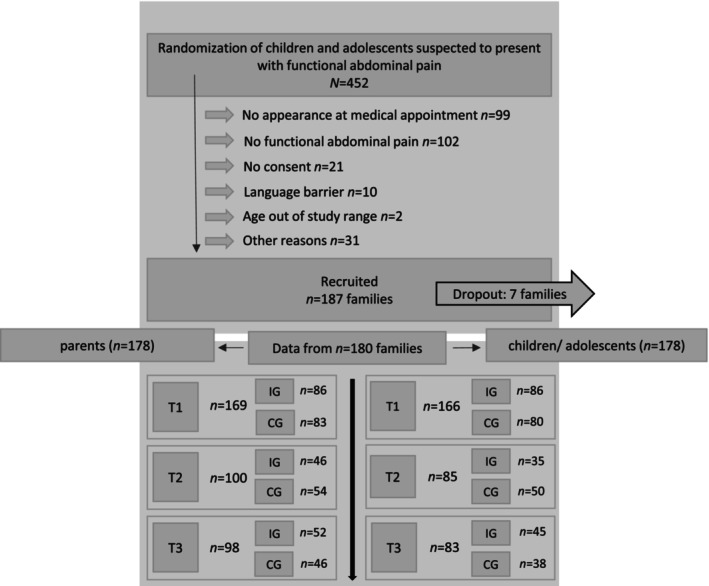
Flowchart of participant retention and exclusions. Overall, *N* = 180 families participated. This corresponds to the number of unique IDs. Each parent–child/adolescent dyad shared the same ID; therefore, the total number of participating children/adolescents and parents may differ, as some assessments were completed only by parents or only by children/adolescents. The upper panel displays reasons for exclusion, including the number of participants affected. The lower panel displays overall participation at each assessment, stratified by group affiliation.

In total, *N* = 166 children and adolescents (53.6% female; *M*_age_ = 10.8, SD = 3.31) participated in our study at T_1_, with *N* = 86 (51.8%) randomised to the intervention group (IG). *N* = 33 were young children between 5 and 7 years (54.5% female; *M*_age_ = 6.24, SD = 0.87; IG = 19 (57.6%)) and *N* = 133 were older children between 8 and 17 years (53.4% female; *M*_age_ = 12.0, SD = 2.62; IG = 67 (50.4%)). Of these, *n* = 163 (98.2%) were born in Germany; *n* = 3 (1.8%) in a different country. Of the corresponding parents, *N* = 169 (85.2% female; *M*_age_ = 41.2, SD = 6.36) participated at T_1_, with *N* = 86 (50.9%) in the intervention group. Most parents were born in Germany (*n* = 134, 79.3%), while *n* = 35 (20.7%) were born in a different country. A flowchart illustrates retention and dropouts across the three measurements (Figure 1).

In the IG, 36.1% of children and adolescents and 45.0% of parents reported having visited the website at T_1_ and/or T_2_.

#### 3.1.2 Dropout Analysis

Dropout analysis for the ITT sample showed that *N* = 101 patients were in the *completer* group (*N* = 65 *dropout* after T_1_). After *p*‐value adjustments using the Benjamini‐Hochberg procedure, no systematic group differences were found. Of the participating parents, *N* = 120 were in the *completer* group (*N* = 49 *dropout*). There were significant group differences after *p*‐value adjustments in the abdominal pain knowledge score (*p* = 0.006) and sex (*p* = 0.006). Parents who dropped out after the first assessment scored significantly worse regarding abdominal pain knowledge (*M* = 6.74) compared to *completers* (*M* = 7.93). Additionally, fathers were more likely to drop out after the first measurement (*n* = 16, 64.0%) than to complete the study (*n* = 9, 36.0%), in contrast to mothers (*dropout*: *n* = 33, 22.9%; *completer*: *n* = 111, 77.1%).

The PP sample comprised *N* = 97 *completer* patients (*N* = 63 *dropout*). No group differences were observed after *p*‐value adjustments. *N* = 116 parents completed at least two assessments (*N* = 47 dropout). A significant group difference after *p*‐value adjustments was observed for abdominal pain knowledge (*p* = 0.023), again showing that *completers* performed significantly better (*M* = 7.96) than those who dropped out (*M* = 6.83).

Moreover, there were significant differences regarding pain disability (PPDI; *t*(77.9) = −3.62, *p* < 0.001) with visitors showing significantly lower disability scores (*M* = 26.6) than IG participants who did not visit the website (*M* = 35.4). No significant differences were found for pain severity (API) between website visitors and non‐visitors of the IG (*t*(66.5) = 0.042, *p* = 0.967). Age of the IG participants did also not affect whether the website was visited or not (*t*(80.9) = 0.05, *p* = 0.666).

### 3.2 Intention‐To‐Treat (ITT) Analysis

To perform the Intention‐to‐treat analysis, all data were analysed as originally randomised.

#### 3.2.1 ITT Child Outcomes at T1

Group comparisons were calculated for all T_1_‐outcomes (CG and IG). Pooled test statistics and *p*‐values are reported in Table 2. No significant group differences were observed for any outcome at T_1_.

**TABLE 2:** Intention‐to‐treat group comparisons (CG, IG) at T_1_ of all child/adolescent outcomes.

Variable	Test statistic	*p*
Primary outcomes		
Abdominal pain knowledge^c^	−1.05	0.295
Positive self instruction^a^	−0.78	0.434
Social support^a^	3575	0.801
Passive pain coping^a^	3779.5	0.705
Pain‐related disability^b^	1.02	0.311
Total school days missed	3500.5	0.643
Partial school days missed	3478.5	0.541
Secondary outcomes		
Abdominal pain severity^d^	1.29	0.200
Practitioner's evaluation^e^	4054	0.215
Physician‐patient interaction^f^	2168.5	0.473

*Note:* The outcomes passive pain coping, social support, total and partial school days missed, practitioner's evaluation, and physician‐patient interaction were calculated using non‐parametric Mann–Whitney *U*‐tests. All other outcomes were computed using parametric *t*‐tests.

^a^ Subscore of the PPCI‐r, paediatric pain coping inventory‐revised.

^b^ PPDI, paediatric pain disability Index.

^c^ A‐PKQ, abdominal pain knowledge questionnaire (child version, ≥ 8 years).

^d^ API, abdominal pain index.

^e^ Evaluation of children and adolescents by practitioners regarding: communication, understanding, reaction, behaviour.

^f^ FAPI, physician‐patient interaction questionnaire (≥ 8 years).

#### 3.2.2 ITT Child Outcomes at Follow‐Up

**FIGURE 2:**
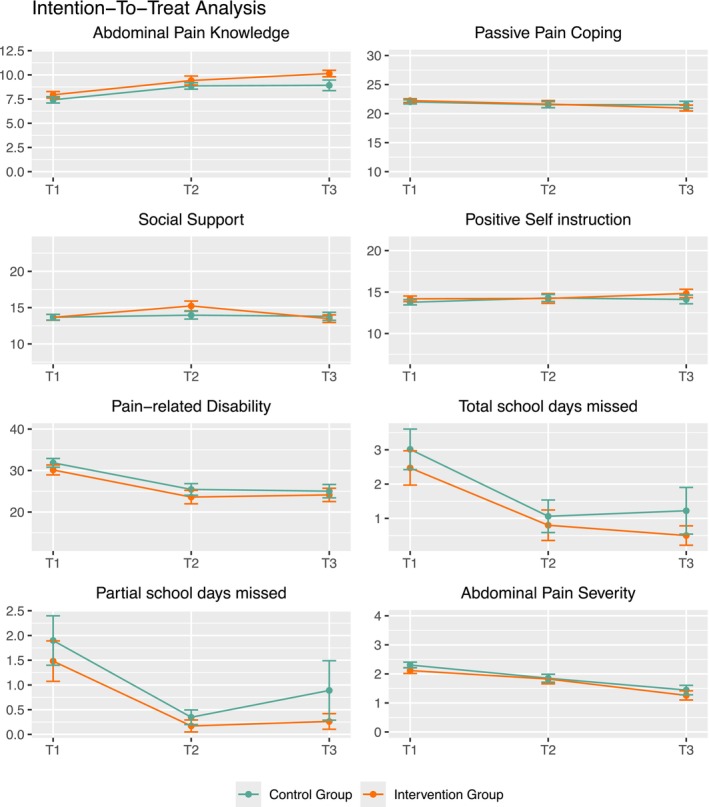
Child/adolescent outcomes in the Intention‐to‐treat analyses over time. The yellow line depicts the intervention group, the green line the control group. The x‐axis depicts assessments (T_1_‐T_3_), while the y‐axis denotes mean values.

Descriptive evaluation showed that patients in the intervention group performed consistently better than the control group across various outcomes, even though none of these differences reached significance in the ITT analysis (see Figure 2 and Table 3). Descriptively, children and adolescents in the IG demonstrated higher levels of abdominal pain knowledge, more frequent searches for social support, more positive self instruction, less pain‐related disability, fewer missed school days, and less abdominal pain compared to the CG in the ITT sample.

**TABLE 3:** Intention‐to‐treat analyses. MLM outcomes for T_1_–T_3_ measurements of child/adolescent outcomes.

	Coefficient	SE	*t*	df_error_	*p*
Passive pain coping^a^					
Intercept	22.67	0.48	47.05	10310.8	< 0.001
Group (KG)	−0.64	0.69	−0.93	11446.8	0.353
Time	−0.47	0.23	−2.07	1730.9	0.039*
Group (KG) × time	0.28	0.32	0.88	2323.3	0.378
Positive self instruction^a^					
Intercept	13.71	0.45	30.50	16462.1	< 0.001
Group (KG)	0.02	0.66	0.03	9128.1	0.980
Time	0.38	0.21	1.83	2971.8	0.068
Group (KG) × time	−0.20	0.31	−0.67	2296.2	0.507
Total school days missed					
Intercept	3.41	0.69	4.97	21315.8	< 0.001
Group (KG)	0.43	0.99	0.43	18966.0	0.664
Time	−1.04	0.34	−3.05	6066.7	0.002*
Group (KG) × time	−0.01	0.49	−0.02	6662.8	0.988
Abdominal pain severity^c^					
Intercept	2.51	0.14	17.47	1903.0	< 0.001
Group (KG)	0.13	0.20	0.64	2812.4	0.525
Time	−0.41	0.07	−6.30	1218.6	< 0.001*
Group (KG) × time	0.02	0.09	0.20	1480.6	0.841
Social support^a^					
Intercept	13.91	0.51	27.54	11987.2	< 0.001
Group (KG)	−0.22	0.73	−0.30	12133.6	0.766
Time	−0.10	0.22	−0.46	1527.0	0.664
Group (KG) × time	0.19	0.32	0.59	1884.4	0.558
Pain‐related disability^b^					
Intercept	32.51	1.62	20.06	10225.2	< 0.001
Group (KG)	1.70	2.34	0.73	8669.2	0.469
Time	−2.89	0.82	−3.54	1716.3	< 0.001*
Group (KG) × time	−0.27	1.16	−0.23	2322.9	0.818
Partial school days missed					
Intercept	1.98	0.56	3.54	22059.5	< 0.001
Group (KG)	0.33	0.81	0.41	21316.8	0.683
Time	−0.60	0.28	−2.13	6584.8	0.033*
Group (KG) × time	0.00	0.41	0.00	6891.3	1.00
Abdominal pain knowledge^d^					
Intercept	7.01	0.44	15.79	7286.5	< 0.001
Group (KG)	−0.20	0.62	−0.32	11508.4	0.749
Time	0.95	0.22	4.33	1168.8	< 0.001*
Group (KG) × time	−0.22	0.31	−0.73	1947.9	0.469

* Significant *p*‐value.

^a^ Subscore of the PPCI‐r, paediatric pain coping inventory‐revised.

^b^ PPDI, paediatric pain disability index.

^c^ API, abdominal pain index (secondary outcome).

^d^ A‐PKQ, abdominal pain knowledge questionnaire (child version, ≥ 8 years).

MLMs were calculated for each outcome measured at all assessments (T_1_–T_3_). Pooled estimates are reported in Table 3. No significant interaction effects were found. However, significant negative time effects were observed for passive pain coping, pain‐related disability, total school days missed, partial school days missed, and abdominal pain. These effects indicate that disabilities and impairments decreased over time, independent of group membership. Additionally, a significant positive effect of time was observed for abdominal pain knowledge, which improved over the course of the study, independent of group membership.

#### 3.2.3 ITT Parent Outcomes at T1

Groups were compared for all T_1_ outcomes (CG vs. IG), but no significant group differences were found (see Table 4). For parent data, a further *t*‐tests testing group differences in abdominal pain knowledge (A‐PKQ) regarding birthplace (*n* = 35 born abroad; *n* = 134 born in Germany) was calculated. No significant differences were found between groups (*U* = 34, *p* = 358).

**TABLE 4:** Intention‐to‐treat group comparisons (CG, IG) for T_1_ measurements of all parent outcomes.

Variable	Test statistic	*p*
Primary outcomes		
Abdominal pain knowledge^a^	3938	0.370
Solicitousness^b^	−0.41	0.682
Distraction^b^	−1.53	0.129
Discouragement^b^	3377.5	0.402
Secondary outcomes		
Physician‐parent interaction^c^	4225.5	0.081
Practitioner's evaluation^d^	3769	0.717
Confidence pain handling	4117	0.177

*Note:* The outcomes abdominal pain knowledge, discouragement, confidence pain handling, physician‐parent interaction, and practitioner's evaluation were calculated using non‐parametric Mann–Whitney *U*‐tests. The remaining two outcomes were analysed by parametric *t*‐tests.

^a^ A‐PKQ, abdominal pain knowledge questionnaire (parent version).

^b^ ISEV‐E, pain‐related parent behaviour inventory.

^c^ FAPI, physician‐parent interaction questionnaire.

^d^ Evaluation of parents by practitioners regarding: communication, understanding, reaction, behaviour.

#### 3.2.4 ITT Parent Outcomes at Follow‐Up

**FIGURE 3:**
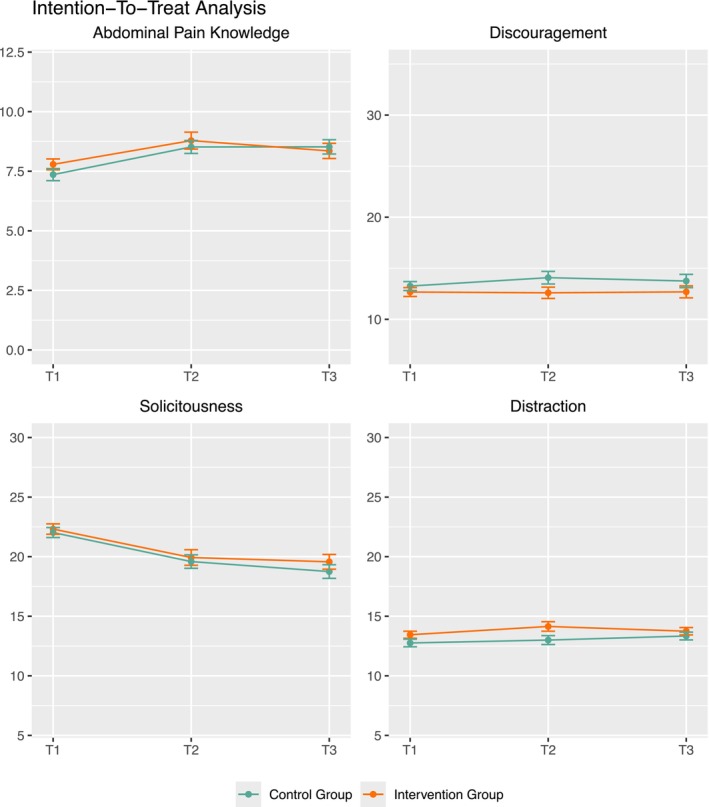
Parent outcomes in the Intention‐to‐treat analyses over time. The yellow line depicts the intervention group, the green line the control group. The x‐axis depicts assessments (T_1_–T_3_), the y‐axis denotes mean values. For the outcome “distraction”, a significant group effect emerged over time (*p* = 0.042). The IG demonstrated significantly more distraction behaviour (*M* = 13.65) compared to the CG (*M* = 13.06).

The descriptive evaluation of all parent outcomes revealed that the intervention group performed slightly better than the control group in the ITT analysis (see Figure 3). Specifically, the intervention group demonstrated more abdominal pain knowledge, less discouraging behaviour, and more distraction in response to their children's pain.

For each outcome, MLMs were calculated using the imputed datasets. Pooled estimates are reported in Table 5. No significant interaction effects were observed. Over time, a significant group effect for distraction emerged, indicating that the intervention group (*M* = 13.65) exhibited more distraction behaviour over time compared to the control group (*M* = 13.06). Significant time effects were found for abdominal pain knowledge, with knowledge improving over time regardless of group membership, and solicitous behaviour decreasing over time.

**TABLE 5:** Intention‐to‐treat analyses. MLMs outcomes for T_1_–T_3_ measurements of parent outcomes.

	Coefficient	SE	*t*	df_error_	*p*
Abdominal pain knowledge^a^					
Intercept	7.53	0.30	24.91	29908.7	< 0.001
Group (KG)	−0.47	0.43	−1.09	47502.7	0.277
Time	0.31	0.12	2.48	10403.0	0.013*
Group (KG) × time	0.13	0.18	0.74	11387.6	0.460
Solicitousness^b^					
Intercept	23.56	0.53	44.44	46735.7	< 0.001
Group (KG)	−0.26	0.76	−0.35	78521.2	0.730
Time	−1.33	0.20	−6.73	11093.5	< 0.001*
Group (KG) × time	−0.06	0.29	−0.20	9495.5	0.841
Discouragement^b^					
Intercept	12.55	0.53	23.76	69162.8	< 0.001
Group (KG)	0.36	0.76	0.47	89733.7	0.635
Time	0.16	0.20	0.80	17215.6	0.425
Group (KG) × time	0.19	0.29	0.65	11939.5	0.515
Distraction^b^					
Intercept	13.15	0.37	35.41	53690.0	< 0.001
Group (KG)	−1.08	0.53	−2.04	130500.0	0.042*
Time	0.27	0.16	1.74	15470.0	0.081
Group (KG) × time	0.32	0.23	1.39	19770.0	0.166

* Significant *p*‐value.

^a^ A‐PKQ, abdominal pain knowledge questionnaire.

^b^ ISEV‐E, Inventar zum schmerzbezogenen Elternverhalten (pain‐related parent behaviour inventory).

### 3.3 Per‐Protocol (PP) Analysis

In the PP analysis, group comparisons were made between participants who reported visiting the website at T_1_ and/or T_2_ (IG; Parents: *n* = 76; Children: *n* = 60). This group was compared to participants who visited the website either not at all or only at T_3_ (CG; Parents: *n* = 93, Children: *n* = 106). Moreover, CG participants who had been exposed to the website were excluded from the analysis.

#### 3.3.1 PP Child Outcomes at T1

**FIGURE 4:**
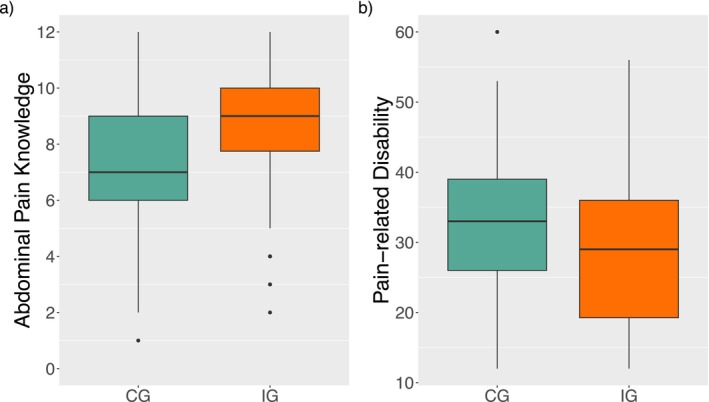
Boxplots of child/adolescent Per‐protocol analyses. Significant group differences were observed at T_1_ for patient knowledge and pain‐related disability outcomes. At the first measurement (T_1_) the group that visited the website (IG; orange block) achieved significantly higher knowledge scores (a: *P* = 0.034; *Mdn* = 9.0) and reported significantly less pain‐related disability (b: *P* = 0.020; *M* = 28.4) compared to the group that did not visit the website (CG; green block; *Mdn* = 7.0, *M* = 32.4 respectively).

Group comparisons were conducted for all T_1_ variables (CG and IG). Pooled test statistics and *p*‐values are reported in Table 6. Significant differences between the CG and IG were observed at the first measurement for abdominal pain knowledge (*U* = 2470, *p* = 0.034), with the IG showing higher pooled median knowledge scores (*Mdn* = 9.0) compared to CG (*Mdn* = 7.0). Additionally, a significant group difference was found for pain‐related disability (*t*(155.8) = 2.34, *p* = 0.020). Pooled mean values reveal a lower frequency of pain‐related disability in those who visited the website (*M* = 28.4) compared to the control group who did not visit the website (*M* = 32.4). Significant effects are visualised in Figure 4.

**TABLE 6:** Per‐protocol group comparisons (CG, IG) at T_1_ of all child/adolescent outcomes.

Variable	Test statistic	*p*
Primary outcomes		
Abdominal pain knowledge^a^	2470	0.034*
Passive pain coping^b^	2918	0.598
Social support^b^	2893	0.540
Positive self instruction^b^	−1.13	0.258
Total school days missed	2806	0.343
Partial school days missed	2769.5	0.236
Pain‐related disability^c^	2.34	0.020*
Secondary outcomes		
Abdominal pain severity^d^	1.26	0.209
Practitioner's evaluation^e^	3102	0.924
Physician‐patient interaction^f^	2081.5	0.756

*Note:* The outcomes passive pain coping, social support, total school days missed, partial school days missed, practitioner's evaluation, abdominal pain knowledge, and physician‐patient Interaction were computed using the non‐parametric Mann–Whitney *U*‐test. All other outcomes were analysed by parametric *t*‐tests.

* Significant *p*‐value.

^a^ A‐PKQ, abdominal pain knowledge questionnaire (child version, ≥ 8 years).

^b^ Subscore of the PPCI‐r, paediatric pain coping inventory‐revised.

^c^ PPDI, paediatric pain disability index.

^d^ API, abdominal pain index.

^e^ Evaluation of children and adolescents by practitioners regarding: communication, understanding, reaction, behaviour.

^f^ FAPI, physician‐patient interaction questionnaire (≥ 8 years).

#### 3.3.2 PP Child Outcomes at Follow‐Up

**FIGURE 5:**
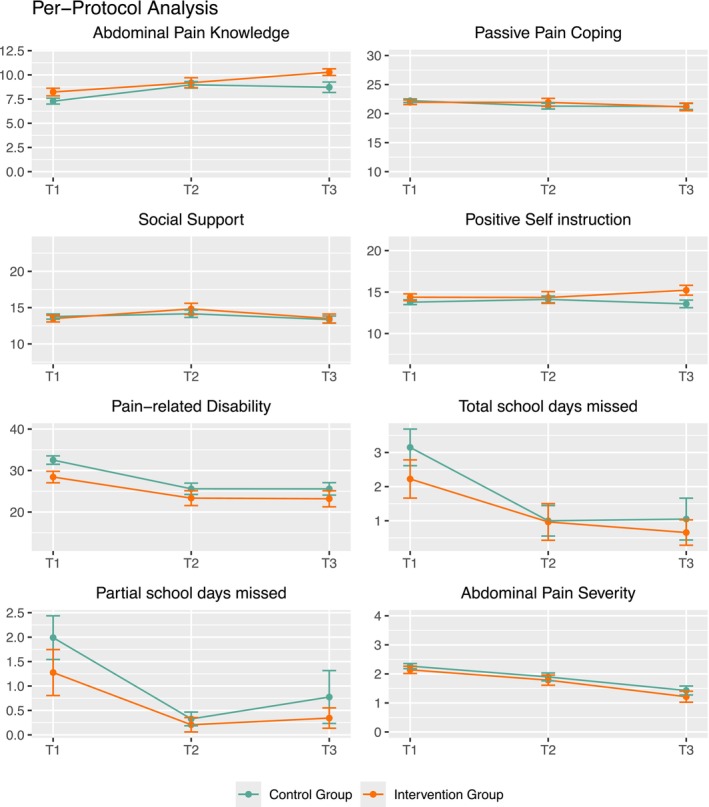
Child/adolescent outcomes in the Per‐protocol analyses over time. The yellow line depicts the intervention group, the green line the control group. The x‐axis depicts assessments (T_1_–T_3_), and the y‐axis denotes mean values. For “pain disability”, a significant group effect emerged (*p* = 0.045) with significantly lower pain‐related disability in the IG (*M* = 25.66) compared to the CG (*M* = 30.71).

Descriptive evaluation showed that the intervention group performed consistently better than the control group in various outcomes, although only pain‐related disability reached significance in the PP analysis (compare Figure 5 and Table 7). Descriptively, children and adolescents in the IG demonstrated greater abdominal pain knowledge, more frequent searches for social support, more positive self instruction, less pain‐related disability, fewer missed school days, and less abdominal pain in the PP analysis.

**TABLE 7:** Per‐protocol analyses. MLM outcomes for T_1_‐T_3_ measurements of all child/adolescent outcomes.

	Coefficient	SE	*t*	df_error_	*p*
Passive pain coping^a^					
Intercept	22.30	0.57	39.08	92325.0	< 0.001
Group (CG)	0.13	0.72	0.18	34416.4	0.860
Time	0.26	0.25	−1.02	5965.6	0.309
Group (CG) × time	−0.12	0.33	−0.36	4702.9	0.722
Positive self instruction^a^					
Intercept	13.90	0.53	26.16	137200.0	< 0.001
Group (CG)	−0.04	0.68	−0.06	23390.0	0.955
Time	0.42	0.23	1.80	9694.0	0.072
Group (CG) × time	−0.40	0.31	−1.30	3871.0	0.195
Total school days missed					
Intercept	2.97	0.84	3.53	82832.4	< 0.001
Group (CG)	1.23	1.07	1.14	21997.6	0.253
Time	−0.81	0.41	−1.95	8214.9	0.051
Group (CG) × time	−0.42	0.55	−0.77	3533.0	0.437
Abdominal pain severity^c^					
Intercept	2.50	0.17	14.62	2947.0	< 0.001
Group (CG)	0.13	0.21	0.61	4141.7	0.545
Time	−0.42	0.07	−5.63	1991.9	< 0.001*
Group (CG) × time	0.03	0.09	0.26	2235.7	0.794
Social support^a^					
Intercept	13.65	0.58	23.47	120000.0	< 0.001
Group (CG)	0.34	0.74	0.46	4240.0	0.644
Time	0.00	0.23	0.01	5697.0	0.989
Group (CG) × time	−0.12	0.31	−0.40	4129.0	0.693
Pain‐related disability^b^					
Intercept	30.24	1.92	15.76	60930.7	< 0.001
Group (CG)	4.92	2.46	2.00	15669.5	0.045*
Time	−2.28	0.92	−2.49	5267.4	0.013*
Group (CG) × time	−1.10	1.20	−0.92	3722.7	0.358
Partial school days missed					
Intercept	1.65	0.69	2.41	99314.7	0.016
Group (CG)	0.84	0.88	0.96	26610.1	0.337
Time	−0.47	0.34	−1.37	10835.6	0.171
Group (CG) × time	−0.22	0.45	−0.49	5309.3	0.622
Abdominal pain knowledge^d^					
Intercept	7.37	0.51	14.34	45331.6	< 0.001
Group (CG)	−0.76	0.67	−1.13	8597.5	0.257
Time	0.86	0.24	3.54	3425.9	0.001*
Group (CG) × time	−0.07	0.33	−0.22	1577.1	0.826

* Significant *p*‐value.

^a^ Subscore of the PPCI‐r: paediatric pain coping inventory‐revised.

^b^ PPDI, paediatric pain disability index.

^c^ API, abdominal pain index (secondary outcome).

^d^ A‐PKQ, abdominal pain knowledge questionnaire (child version, ≥ 8 years).

MLMs were calculated for each outcome measured at all assessments (T_1_–T_3_). No significant interaction effects were found. A significant group effect emerged for pain‐related disability, showing that the IG (*M* = 25.66; website visited) had significantly lower pain‐related disability scores compared to CG (*M* = 30.71; website not visited) across all assessments. Moreover, a significant time effect was found, indicating a decrease in pain‐related disability over time regardless of group affiliation. A significant time effect was also found for abdominal pain, indicating that disability decreased over time independent of group membership. A further significant time effect was found for abdominal pain knowledge, indicating that knowledge improved over time for all participants (see Table 7).

#### 3.3.3 PP Parent Outcomes at T1

**FIGURE 6:**
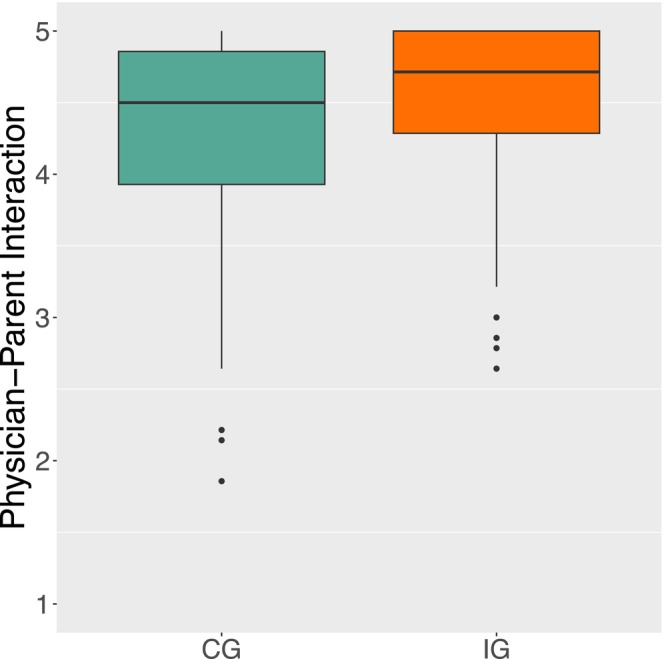
Boxplot of parent Per‐protocol analysis. At the first measurement (T_1_) parents who visited the website (IG; orange block) rated the physician‐parent interaction significantly more positively (*p* = 0.006; *Mdn* = 4.71) than parents who did not visit the website (CG; *Mdn* = 4.5; green block).

Group comparisons were conducted for all T_1_‐outcomes (IG and CG; compare Table 8). Significant group differences were found for the physician‐parent interaction (*U* = 4185.5, *p* = 0.006), with those who visited the website rating the physician‐parent interaction significantly better (*Mdn* = 4.71) than those who did not visit the website (*Mdn* = 4.5; Figure 6).

**TABLE 8:** PP *T*‐tests for T_1_ measurements of all parent outcomes.

Variable	Test statistic	*p*
Primary outcomes		
Abdominal pain knowledge^a^	3811	0.115
Solicitousness^b^	−0.56	0.573
Distraction^b^	−1.57	0.119
Discouragement^b^	2831.5	0.097
Secondary outcomes		
Physician‐parent interaction^c^	4185.5	0.006*
Practitioner's evaluation^d^	3172	0.580
Confidence pain handling	−0.46	0.644

*Note:* The outcomes of practitioner's evaluation, abdominal pain knowledge, physician‐parent interaction, and discouragement were calculated using non‐parametric Mann–Whitney *U*‐tests. The other outcomes were analysed with parametric *t*‐tests.

* Significant *p*‐value.

^a^ A‐PKQ, abdominal pain knowledge questionnaire.

^b^ ISEV‐E, Inventar zum schmerzbezogenen Elternverhalten (pain‐related parent behaviour inventory).

^c^ FAPI, fragebogen zur arzt‐patienten‐interaktion (physician‐parent interaction questionnaire).

^d^ Evaluation of parents by practitioners regarding: communication, understanding, reaction, behaviour.

#### 3.3.4 PP Parent Outcomes at Follow‐Up

**FIGURE 7:**
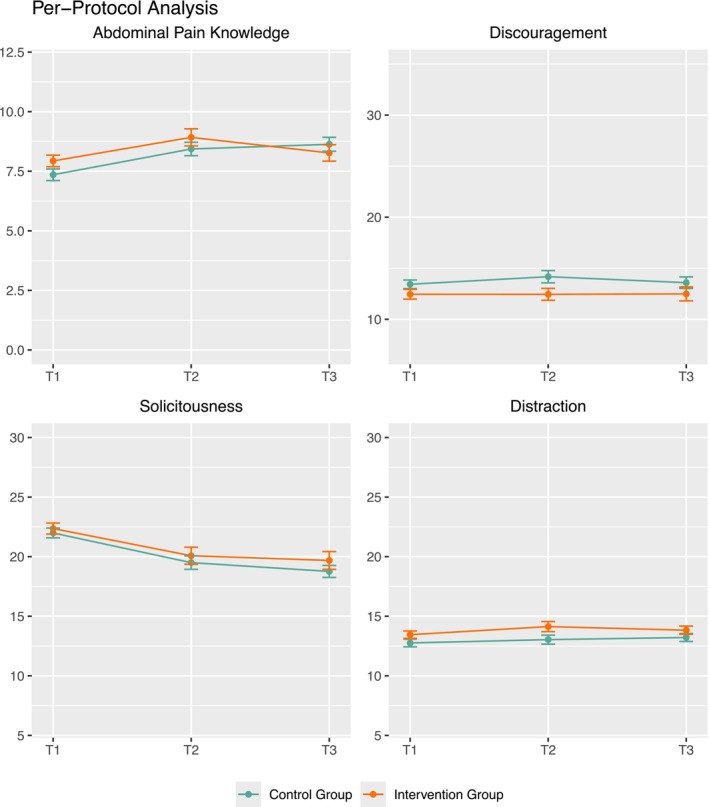
Parent outcomes in the Per‐protocol analyses over time. The yellow line depicts the intervention group, the green line the control group. The x‐axis depicts assessments (T_1_–T_3_), and the y‐axis denotes mean values.

Descriptive evaluation of all parent outcomes revealed that the intervention group outperformed the control group in the PP analysis (see Figure 7). The intervention group showed more abdominal pain knowledge, less discouraging behaviour, and more distraction in response to their children's pain compared to the CG in the PP analysis.

For the MLMs, pooled estimates are reported (Table 9). No significant interaction effects were found. A significant time effect was found for solicitous parent behaviour, indicating that solicitousness decreased over time regardless of group membership.

**TABLE 9:** Per‐protocol analyses. MLMs outcomes for T_1_–T_3_ measurements of all parent outcomes.

	Coefficient	SE	*t*	df_error_	*p*
Abdominal pain knowledge^a^					
Intercept	7.80	0.32	24.17	365700.0	< 0.001
Group (CG)	−0.82	0.44	−1.85	83100.0	0.064
Time	0.22	0.14	1.65	15660.0	0.099
Group (CG) × time	0.26	0.19	1.40	18440.0	0.162
Discouragement^b^					
Intercept	12.28	0.57	21.70	569000.0	< 0.001
Group (CG)	0.92	0.78	1.18	79050.0	0.239
Time	0.19	0.21	0.86	13540.0	0.388
Group (CG) × time	0.08	0.30	0.26	12250.0	0.798
Solicitousness^b^					
Intercept	23.61	0.57	41.50	659700.0	< 0.001
Group (CG)	−0.35	0.78	−0.45	86710.0	0.652
Time	−1.30	0.22	−6.03	15630.0	< 0.001*
Group (CG) × time	−0.14	0.30	−0.46	12330.0	0.643
Distraction^b^					
Intercept	13.18	0.40	32.93	614200.0	< 0.001
Group (CG)	−1.05	0.55	−1.02	134200.0	0.055
Time	0.27	0.17	1.57	32950.0	0.117
Group (CG) × time	0.30	0.24	1.23	29060.0	0.218

* Significant *p*‐value.

^a^ A‐PKQ, abdominal pain knowledge questionnaire.

^b^ ISEV‐E, Inventar zum schmerzbezogenen Elternverhalten (pain‐related parent behaviour inventory).

### 3.4 Exploratory Analyses

#### 3.4.1 Children and Adolescents

Additional MLMs were performed for exploratory purposes. Age and sex were included as variables in the models to explore whether these demographic variables would increase power and explain more variance.

After including age and sex in the models, no further effects were observed compared to the original models (see Supporting Information Tables S1 and S3).

#### 3.4.2 Parents

Exploratory MLMs were also conducted for parent outcomes to examine the effect of including age and sex.

In the ITT analysis, a significant change was observed for distraction. Unlike the MLM analysis that included only the group × time interaction, after adding age and sex to the model, the group effect was no longer significant (*t*(105500.0) = −1.88, *p* = 0.061). However, distraction behaviour decreased with increasing age (*t*(52880.0) = −2.59, *p* = 0.010).

No other changes in group, time, or interaction effects were observed with the inclusion of age and sex in the model (see Supporting Information Tables S2 and S4).

### 3.5 Website Use and Evaluation

#### 3.5.1 Child Use and Evaluation

Of the IG children who participated in the questions about website usage (T_1_: *n* = 83; T_2_: *n* = 34; T_3_: *n* = 41), 60.2% indicated visiting the website at T_1_, 85.3% at T_2_, and 75.6% at T_3_. Self‐reported website visit duration lasting at least 30 min was reported by 48.0% at T_1_, 41.4% at T_2_, and 29.0% at T_3_. An overview of website usage revealed that the sections on abdominal pain and functional abdominal pain were the most frequently visited across all assessments (see Table 10).

**TABLE 10:** Overview of website visits for children/adolescents and parents in the IG at each measurement, presented as percentages.

Website section	Children and adolescents (%)	Parents (%)
T1		
Gastrointestinal tract^a^	72.0	—
Abdominal pain	88.0	76.7
Functional abdominal pain	74.0	68.6
Effective management^b^	48.0	
Nutrition	—	48.4
Support	—	40.7
T2		
Gastrointestinal tract^a^	55.2	—
Abdominal pain	65.5	88.9
Functional abdominal pain	93.1	97.2
Effective management^b^	69.0	
Nutrition	—	63.9
Support	—	66.7
T3		
Gastrointestinal tract^a^	58.1	—
Abdominal pain	77.4	74.4
Functional abdominal pain	71.0	92.3
Effective management^b^	61.3	
Nutrition	—	74.4
Support	—	61.5

^a^ Knowledge about the gastrointestinal tract is part of only the child and adolescent area of the website, and therefore not applicable to parents.

^b^ The section about effective management of functional abdominal pain is split into two domains (“nutrition” and “support”) in the parent area of the website, and thus are reported individually.

Patients were asked at the final measurement to evaluate the website and provide feedback on the website experience. The website was rated by awarding 1–5 stars (1 = “*unsatisfactory*”; 5 = “*very good*”). Participants rated the website positively with a mean score of 4.00 out of 5 stars (SD = 0.97). Of the patients who participated, 61.3% stated they had implemented the advice provided on the website. The most often mentioned recommendations were “distraction“, “concentrate on other things“, “exercise”, and “relaxation”. Additionally, 74.2% of children found the information on the website to be mostly or entirely comprehensible. They emphasised that they particularly liked the “clear and understandable explanations” and the multimedia elements. Almost no negative feedback was given, except for two children who did not like the amount of text in certain areas or the occasional use of “child‐like” presentations or explanations.

Only *N* = 4 patients of the CG claimed at T_3_ to have heard of the website.

#### 3.5.2 Parent Use and Evaluation

In total (T_1_: *n* = 84; T_2_: *n* = 42; T_3_: *n* = 51), 82.6% of parents in the IG reported visiting the website at T_1_, 78.3% at T_2_, and 75.0% at T_3_. Of those who visited the website, 53.5% at T_1_, 52.7% at T_2_, and 59.0% at T_3_ self‐reported visiting for at least 30 min. The website sections on abdominal pain and functional abdominal pain were the most popular across all measurements (see Table 10).

At T_3_, parents rated the website as good to very good, with a mean of 4.23 of 5 stars (SD = 0.93). Of the parents who participated in our survey, 87.2% stated that they implemented the advice given on the website. The most frequently mentioned helpful tips were “distraction” and “exercise.” Regarding the clarity of the website's content, 89.5% of parents indicated that they understood most or all of the information. Notably, parents highlighted the “child‐friendly explanations” and “vivid presentation” of information, including the multimedia elements. Negative feedback was rare, with most parents answering “no” when asked if there were aspects they disliked. Only a few comments mentioned technical problems. Similarly, 92.0% of parents answered “none” when asked about suggestions for improvement.

Only *N* = 4 parents in the CG reported being aware of the website at T_3_.

## 4 Discussion

The present RCT investigated the effect of a newly developed educational website about functional abdominal pain (FAP; https://meine‐bauchstelle.com) on pain knowledge, behaviour, and characteristics in a sample of paediatric patients suffering from FAP and their parents. In addition to an Intention‐to‐treat (ITT) analysis, in which data were analysed as originally randomised, a Per‐protocol (PP) analysis was conducted.

In contrast to the ITT, the PP analysis revealed positive effects on knowledge and health behaviour. Patients who visited our educational website prior to their first appointment at paediatric gastroenterology clinics demonstrated greater initial (T_1_) knowledge of FAP compared to those who did not. This effect, however, was not stable over assessments—potentially due to the temporary integration of website content into one's cognitive framework (Zheng et al. 2024), facilitating recall at the first assessment. Presumably, website use—combined with physician consultation—initially reinforced knowledge but diminished over time as the physician's visit became the dominant factor for both groups regardless of website usage. Continued engagement with website content might strengthen these effects through memory consolidation (Haubrich and Nader 2018; Kida 2020).

Moreover, website visitors experienced significantly less pain‐related disability both initially and across all assessments compared to non‐visitors. A partial contradiction of assuming no significant initial differences but a favourable trajectory in the IG. Similarly, when comparing IG website visitors and non‐visitors, non‐visitors experienced significantly more pain disability than visitors. These results suggest that the website offered helpful information, but the patients felt too disabled to utilise it. It may help to allow parents to support their children while using the website. Participants experiencing more pain disability fell out of the ‘visitor’—group in the PP analysis. This might partly explain higher PPDI scores at T_1_ in the non‐visitor group. Behavioural changes in visitors may have occurred earlier than anticipated, consistent with improvements observed for adult chronic pain education (Sidiq et al. 2024). No further significant effects were found for other health behaviour outcomes (e.g., pain coping, school absenteeism), abdominal pain symptoms, or physician‐patient interaction. However, our study included a strong control intervention: all patients, including those in the CG, visited a paediatric gastroenterologist before their first assessment. This high‐quality care likely contributed to improvements in FAP symptoms which is reflected by the improvements in most outcomes over time, regardless of group membership. Given the effectiveness of this medical consultation, small website effects can be expected but difficult to translate into significant long‐term group differences. Furthermore, the physician consultation may have improved pain by enhancing pain self‐efficacy through effective communication with the physician or joint decision‐making (Lemos et al. 2024). Stronger interventions beyond education may be necessary, evidenced by previous educational interventions for children with FAP and adults with chronic pain (Goldstein et al. 2025; Laerkner et al. 2023), which showed little impact on pain behaviour, characteristics, or knowledge (Geneen et al. 2015; Goldstein et al. 2025; Laerkner et al. 2023; Pas et al. 2020). Other interventions, however, like cognitive behavioural therapy (CBT) produce significantly better health outcomes than knowledge transfer alone (Darnall et al. 2021; Levy et al. 2010).

Contrary to our hypothesis, no significant knowledge gains were observed among parents. However, other research indicates that educational interventions do not always measurably improve caregiver knowledge (Fechner et al. 2024). Notably, the the parents who left the study early likely would have benefited most from additional knowledge. For our secondary outcome, parents who visited the website rated their physician interaction significantly better than those who did not. Perhaps parents who accessed the website beforehand felt better equipped with the knowledge they acquired and less overwhelmed by the information received during consultation. This result supports other studies showing that better engagement, knowledge, and coping behaviours contribute to improved health, particularly in physician‐parent interactions (Greene et al. 2013, 2015). Providing education before a physician's appointment may improve perceptions of the consultation, health outcomes, and self‐management (Kelley et al. 2014; Orom et al. 2018). This effect, however, was not observed in the ITT analysis, suggesting that merely granting website access before the physician's appointment seems insufficient to improve perceptions of the consultation. Active engagement with the content is key.

In the ITT analysis, the IG demonstrated significantly more distracting behaviour towards their child's pain over time compared to the control group. However, this effect was not significant in the PP analysis, suggesting that the website did not directly cause this behaviour change. Exploratory analyses revealed that when increasing model power, younger parents tended to use distraction strategies for their child's pain more frequently than older parents.

Contrary to our hypothesis, neither the ITT nor the PP analyses revealed significant interaction effects. We suspect that repeated website visits would reinforce knowledge and enhance primary and secondary outcomes for the IG compared to the CG, leading to significant interaction effects. However, significant time effects were observed for pain knowledge, behaviour, and characteristics in both ITT and PP analyses, all of which improved over time.

Although website engagement was lower than anticipated, feedback from visitors was highly positive. Most users spent at least half an hour on the site, enabling them to provide well‐informed feedback. They rated their website experience as good to very good, emphasising its effectiveness in content delivery, structure, and comprehensibility.

Our website was designed for patients experiencing functional abdominal pain (FAP). Chronic abdominal pain is one of the most prevalent paediatric chronic pain disorders (Koen Vermeijden et al. 2025). Often, chronic pain starts in one location and spreads to other regions over time (Senger‐Carpenter et al. 2022). Several chronic pain management techniques on our website (e.g., adequate sleep and exercise) are transferable to other pain conditions. However, many families encounter the concept of chronic pain for the first time upon diagnosis. They likely feel taken more seriously when offered a website tailored to the specific pain condition. Such a resource can help build trust and reduce uncertainty by providing detailed information about the chronic pain type without overwhelming the website visitor.

## 5 Limitations

Since not all IG participants who had access to the website used it, a PP analysis was performed. While most group differences were found in the PP analysis, statistical power was reduced due to the smaller IG sample size. Descriptive trends suggest that both children and parents in the IG performed better than those in the CG. Website exposure may have contributed to outcome improvements, though these effects did not reach statistical significance. However, PP analyses introduce potential biases, complicating group comparisons.

Our study samples may be biased towards more educated parents; parents who dropped out of the study had lower knowledge, a potential reflection of negative self‐perception or feeling overwhelmed by their child's pain. The relationship between low therapy adherence and less knowledge has been investigated in other fields (Awwad et al. 2015; Pourhabibi et al. 2022). Additionally, Fathers are underrepresented in child research and are more likely to drop out of studies, which was also observed in our study (Parent et al. 2017; Schulz et al. 2023). These dropout patterns should be considered when interpreting our results.

Due to our study design, physicians were blinded to participants' group affiliation and could not give additional website recommendations. It is likely that a physician's endorsement would have increased website usage, potentially resulting in significant interaction effects. Physician advice is often more influential than that of other professionals (Gerber et al. 2014), and mothers in particular tend to trust their advice (Hwang et al. 2017), promoting treatment adherence (Flecia and Mohd 2023). Therefore, with parent support, physicians may play a central role in facilitating patients' website engagement—particularly patients whose engagement is limited by their high pain‐related disability. In the current study, website usage was self‐reported, not tracked. For a precise and objective dosage‐effect analysis, a log‐in to the website would have been necessary. To reduce barriers for patients visiting the website, a log‐in was avoided. While this reduces precision of usage analyses, this offers the decisive advantage that the study took place under real‐life conditions.

Moreover, providing a workbook guiding through the website content might be a helpful addition. In a similar study, evaluating efficacy of a website about chronic headache, providing similar material was shown to be an efficient tool (Goldstein et al. 2025).

## 6 Practical Implications and Future Directions

This website, developed using a participatory approach, offers a valuable contribution to the treatment of children and adolescents with functional abdominal pain. It can serve as an additional aid in routine clinical care, where time constraints often limit opportunities for in‐depth education, by allowing healthcare providers to refer patients to the website for further explanations and support. Given the high comorbidity of fructose and lactose malabsorption with FAP (Garg et al. 2017; Schnedl et al. 2023), future studies could investigate whether website effectiveness differs by malabsorption status to characterise the needs of this subgroup and inform clinical practice. The same applies to psychiatric comorbidities (e.g., anxiety or depression), as the website may also support pain education within psychotherapy. Furthermore, a recent study revealed that school‐based educational interventions are a promising strategy for managing paediatric pain (Ojha et al. 2024). If our website was implemented in schools, it could serve as a preventive measure against the development of functional abdominal pain in children and adolescents. While no association between migration background and A‐PKQ knowledge was observed in the present study, including more sociodemographic variables (e.g., parental education level) in future studies may help tailor interventions to different target populations.

## 7 Conclusions

Our educational website about functional abdominal pain for children, adolescents, and parents (https://meine‐bauchstelle.com) was effective in enhancing knowledge in children and adolescents, reducing pain‐related disability, and improving physician‐parent interactions. Although the long‐term effects of website access in the context of our study were modest, this low‐threshold intervention still led to positive outcomes. Integrating it into clinical practice could catalyse its effects.

## Author Contributions

All authors listed have contributed substantially to the manuscript. J.W. and V.N. were responsible for the conception and design of the study. Data were collected and managed in the three participating clinics (T.B., N.C.S., J.B., V.N.). V.N., C.H., L.‐M.R., L.E., G.H., C.B.‐E. and J.W. contributed to the data analysis and interpretation of results. V.N. drafted the manuscript, which all authors critically revised for intellectual content. Each author has read the final manuscript and approved its submission.

## Funding

This study was funded by the Innovation Committee of the Federal Joint Committee (grant number: 01VSF19024).

## Conflicts of Interest

Conflicts of interest are declared by C.V. who was employed by the health insurance company Techniker Krankenkasse, and S.B., who is a member of advisory boards at Danone Deutschland and Infectopharm. He has received speaker's honoraria from Falk Foundation, Infectopharm, Nutricia and Nestle NNI. The remaining authors declare no conflicts of interest.

## Supporting information


**Data S1:** ejp70249‐sup‐0001‐DataS1.docx.
