# Supplementary material for: A Randomised Controlled Trial Testing the Efficacy of an Educational Website About Functional Abdominal Pain for Children and Adolescents
Source: Eur J Pain. 2026 Mar 18;30(3):e70249. doi: 10.1002/ejp.70249 (PMC12997388; doi:10.1002/ejp.70249)
Supplement: Supplementary file 1 — Data S1: ejp70249‐sup‐0001‐DataS1.docx. [file EJP-30-0-s001.docx]

# Supplementary Materials

## Supplementary Material S1

**Table S1**

*Exploratory Intention-to-treat analyses. MLMs outcomes including predictors sex and age for T_1_-T_3_ measurements of all child/adolescent outcomes.*

|  | **Coefficient** | **SE** | ***t*** | **df_error_** | ***p*** |  | **Coefficient** | **SE** | ***t*** | **df_error_** | ***p*** |
| --- | --- | --- | --- | --- | --- | --- | --- | --- | --- | --- | --- |
| **Passive Pain Coping^a^** | | | | | | **Social Support^a^** | | | | | |
| Intercept | 20.74 | 0.85 | 24.39 | 16320.4 | <.001 |  | 18.91 | 0.87 | 21.73 | 11001.8 | <.001 |
| Group (CG) | -0.59 | 0.68 | -0.86 | 10941.3 | .391 |  | -0.16 | 0.68 | -0.23 | 9823.8 | .819 |
| Time | -0.48 | 0.23 | -2.14 | 1734.7 | **.032*** |  | -0.06 | 0.22 | -0.25 | 1514.1 | .801 |
| Sex (female) | 1.06 | 0.44 | 2.40 | 11321.3 | **.017*** |  | 0.67 | 0.45 | 1.49 | 20098.3 | .136 |
| Age | 0.13 | 0.06 | 2.00 | 49461.6 | **.046*** |  | -0.50 | 0.07 | -7.65 | 28717.5 | **<.001*** |
| Group (CG) x time | 0.30 | 0.32 | 0.93 | 2327.3 | .355 |  | 0.14 | 0.32 | 0.44 | 1869.3 | .661 |
| **Positive Self instruction^a^** | | | | | | **Pain-related Disability^b^** | | | | | |
| Intercept | 12.60 | 0.81 | 15.50 | 15343.4 | <.001 |  | 22.55 | 2.58 | 8.74 | 5852.1 | <.001 |
| Group (CG) | 0.01 | 0.66 | 0.01 | 9183.1 | .994 |  | 1.64 | 2.29 | 0.72 | 7908.1 | .474 |
| Time | 0.37 | 0.21 | 1.78 | 3049.9 | .075 |  | -2.98 | 0.81 | -3.70 | 1669.8 | **<.001*** |
| Sex (female) | -0.08 | 0.41 | -0.19 | 32967.9 | .852 |  | -0.79 | 1.23 | -0.64 | 10177.9 | .520 |
| Age | 0.11 | 0.06 | 1.79 | 47313.3 | .073 |  | 0.98 | 0.18 | 5.37 | 10485.2 | **<.001*** |
| Group (CG) x time | -0.19 | 0.31 | -0.63 | 2310.4 | .529 |  | -0.19 | 1.15 | -0.16 | 2275.8 | .871 |
| **Total school days missed** | | | | | | **Partial school days missed** | | | | | |
| Intercept | 0.94 | 1.04 | 0.90 | 10046.5 | .366 |  | 0.27 | 0.82 | 0.33 | 8966.7 | .742 |
| Group (CG) | 0.36 | 0.98 | 0.37 | 17805.9 | .714 |  | 0.30 | 0.80 | 0.38 | 20668.5 | .707 |
| Time | -1.04 | 0.34 | -3.07 | 6105.0 | **.002*** |  | -0.61 | 0.28 | -2.17 | 6427.8 | **.030*** |
| Sex (female) | -0.86 | 0.49 | -1.76 | 7834.2 | .079 |  | -0.34 | 0.37 | -0.91 | 5914.2 | .364 |
| Age | 0.28 | 0.07 | 3.86 | 11554.4 | **<.001*** |  | 0.18 | 0.05 | 3.26 | 8338.1 | **.001*** |
| Group (CG) x time | 0.03 | 0.49 | 0.06 | 6823.3 | .955 |  | 0.02 | 0.41 | 0.04 | 6742.8 | .968 |
| **Abdominal Pain severity^c^** | | | | | | **Abdominal Pain Knowledge**^d^ | | | | | |
| Intercept | 2.01 | 0.25 | 7.98 | 2296.1 | <.001 |  | 5.70 | 0.98 | 5.80 | 5069.2 | <.001 |
| Group (CG) | 0.13 | 0.20 | 0.62 | 2744.7 | .533 |  | -0.14 | 0.62 | -0.23 | 11425.6 | .818 |
| Time | -0.41 | 0.07 | -6.38 | 1224.4 | **.001*** |  | 0.94 | 0.22 | 4.30 | 1167.9 | **<.001*** |
| Sex (female) | -0.03 | 0.12 | -0.23 | 5182.8 | .821 |  | 0.24 | 0.39 | 0.62 | 7260.9 | .536 |
| Age | 0.05 | 0.02 | 2.60 | 5058.2 | **.009*** |  | 0.10 | 0.07 | 1.35 | 5250.4 | .177 |
| Group (CG) x time | 0.02 | 0.09 | 0.24 | 1489.3 | .811 |  | -0.22 | 0.31 | -0.72 | 1953.4 | .472 |

*Significant *p*-value

^a^Subscore of the PPCI-r: Paediatric Pain Coping Inventory-revised

^b^PPDI: Pediatric Pain Disability Index

^c^API: Abdominal Pain Index (Secondary Outcome)

^d^A-PKQ: Abdominal Pain Knowledge Questionnaire (Child Version, ≥ 8 years)

## Supplementary Material S2

**Table S2**

*Exploratory Intention-to-treat analyses. MLMs outcomes including predictors sex and age for T_1_-T_3_ measurements of all parent outcomes.*

|  | **Coefficient** | **SE** | ***t*** | **df_error_** | ***p*** | |  | **Coefficient** | **SE** | ***t*** | **df_error_** | ***p*** | |
| --- | --- | --- | --- | --- | --- | --- | --- | --- | --- | --- | --- | --- | --- |
| **Abdominal Pain Knowledge^a^** | | | | | | **Discouragement^b^** | | | | | | |  |
| Intercept | 6.54 | 1.15 | 5.71 | 12989.4 | <.001 | |  | 11.19 | 2.12 | 5.27 | 11957.0 | <.001 | |
| Group (CG) | -0.44 | 0.43 | -1.02 | 44006.4 | .306 | |  | 0.38 | 0.76 | 0.51 | 91283.9 | .613 | |
| Time | 0.29 | 0.12 | 2.35 | 10959.5 | **.019*** | |  | 0.14 | 0.20 | 0.71 | 17628.6 | .476 | |
| Sex (female) | 0.66 | 0.44 | 1.50 | 2655.1 | .135 | |  | 0.75 | 0.81 | 0.93 | 1515.8 | .354 | |
| Age | 0.01 | 0.02 | 0.43 | 38924.6 | .666 | |  | 0.02 | 0.04 | 0.40 | 50275.1 | .692 | |
| Group (CG) x time | 0.14 | 0.18 | 0.75 | 11334.0 | .453 | |  | 0.19 | 0.29 | 0.66 | 12017.6 | .507 | |
| **Solicitousness^b^** | | | | | | **Distraction^b^** | | | | | | |  |
| Intercept | 22.92 | 2.12 | 10.81 | 10516.7 | <.001 | |  | 15.88 | 1.36 | 11.66 | 18380.0 | <.001 | |
| Group (CG) | -0.32 | 0.76 | -0.42 | 78096.1 | .675 | |  | -0.99 | 0.53 | -1.88 | 105500.0 | .061 | |
| Time | -1.33 | 0.20 | -6.67 | 11447.0 | **<.001*** | |  | 0.28 | 0.16 | 1.77 | 16390.0 | .076 | |
| Sex (female) | -0.57 | 0.83 | -0.69 | 1193.6 | .488 | |  | 0.25 | 0.52 | 0.47 | 3897.0 | .636 | |
| Age | 0.03 | 0.04 | 0.64 | 45193.8 | .525 | |  | -0.07 | 0.03 | -2.59 | 52880.0 | **.010*** | |
| Group (CG) x time | -0.06 | 0.29 | -0.20 | 9582.1 | .843 | |  | 0.31 | 0.23 | 1.34 | 18400.0 | .181 | |

*Significant *p*-value

^a^A-PKQ: Abdominal Pain Knowledge Questionnaire

^b^ISEV-E: Inventar zum schmerzbezogenen Elternverhalten (Pain-related Parent Behavior Inventory)

## Supplementary Material S3

**Table S3**

*Exploratory Per-protocol analyses. MLMs outcomes including predictors sex and age for T_1_-T_3_ measurements of all child/adolescent outcomes.*

|  | **Coefficient** | **SE** | ***t*** | **df_error_** | ***p*** |  | **Coefficient** | **SE** | ***t*** | **df_error_** | ***p*** |
| --- | --- | --- | --- | --- | --- | --- | --- | --- | --- | --- | --- |
| **Passive Pain Coping^a^** | | | | | | **Social Support^a^** | | | | | |
| Intercept | 20.33 | 0.94 | 21.57 | 32063.5 | <.001 |  | 18.93 | 0.93 | 20.32 | 34140.8 | <.001 |
| Group (CG) | 0.29 | 0.72 | 0.41 | 33145.9 | .683 |  | 0.08 | 0.68 | 0.11 | 33522.3 | .911 |
| Time | -0.28 | 0.25 | -1.10 | 5996.9 | .272 |  | 0.06 | 0.23 | 0.28 | 5690.1 | .783 |
| Sex (female) | 0.97 | 0.45 | 2.18 | 18298.5 | **.029*** |  | 0.55 | 0.45 | 1.22 | 23462.8 | .221 |
| Age | 0.13 | 0.07 | 1.96 | 43372.6 | .050 |  | -0.51 | 0.07 | -7.64 | 46913.5 | **<.001*** |
| Group (CG) x time | -0.10 | 0.33 | -0.32 | 4714.1 | .753 |  | -0.18 | 0.30 | -0.60 | 4061.9 | .548 |
| **Positive Self instruction^a^** | | | | | | **Pain-related Disability^b^** | | | | | |
| Intercept | 12.82 | 0.90 | 14.28 | 45886.6 | <.001 |  | 19.20 | 2.82 | 6.81 | 14046.4 | <.001 |
| Group (CG) | 0.01 | 0.68 | 0.02 | 22730.3 | .983 |  | 5.34 | 2.39 | 2.24 | 14905.9 | **.025*** |
| Time | 0.40 | 0.23 | 1.75 | 9817.3 | .081 |  | -2.49 | 0.91 | -2.74 | 5288.4 | **.006*** |
| Sex (female) | -0.08 | 0.43 | -0.19 | 27186.6 | .848 |  | -0.68 | 1.25 | -0.55 | 10055.6 | .586 |
| Age | 0.10 | 0.06 | 1.60 | 44620.9 | .109 |  | 1.04 | 0.19 | 5.64 | 10834.8 | **<.001*** |
| Group (CG) x time | -0.39 | 0.31 | -1.25 | 3948.2 | .210 |  | -0.87 | 1.18 | -0.74 | 3788.0 | .462 |
| **Total school days missed** | | | | | | **Partial school days missed** | | | | | |
| Intercept | 0.28 | 1.20 | 0.235 | 12194.7 | .814 |  | -0.22 | 0.94 | -0.23 | 16803.9 | .817 |
| Group (CG) | 1.25 | 1.07 | 1.17 | 19464.6 | .242 |  | 0.88 | 0.87 | 1.01 | 26071.7 | .312 |
| Time | -0.85 | 0.41 | -2.05 | 8532.1 | **.040*** |  | -0.50 | 0.34 | -1.46 | 10952.5 | .145 |
| Sex (female) | -0.95 | 0.52 | -1.84 | 4824.2 | .066 |  | -0.35 | 0.39 | -0.89 | 5093.4 | .373 |
| Age | 0.29 | 0.07 | 3.97 | 11672.9 | **<.001** |  | 0.19 | 0.06 | 3.38 | 13592.1 | **.001*** |
| Group (CG) x time | -0.34 | 0.55 | -0.61 | 3504.8 | .539 |  | -0.17 | 0.45 | -0.40 | 5366.8 | .697 |
| **Abdominal Pain severity^c^** | | | | | | **Abdominal Pain Knowledge^d^** | | | | | |
| Intercept | 1.92 | 0.27 | 7.07 | 5104.2 | <.001 |  | 6.17 | 1.04 | 5.94 | 5892.2 | <.001 |
| Group (CG) | 0.16 | 0.21 | 0.75 | 4024.1 | .451 |  | -0.70 | 0.68 | -1.03 | 8000.8 | .303 |
| Time | -0.42 | 0.07 | -5.72 | 1961.6 | **<.001*** |  | 0.85 | 0.24 | 3.48 | 3437.9 | **.001*** |
| Sex (female) | -0.02 | 0.13 | -0.12 | 5262.4 | .903 |  | 0.13 | 0.40 | 0.32 | 8050.6 | .752 |
| Age | 0.05 | 0.02 | 2.76 | 4685.7 | **.006*** |  | 0.09 | 0.07 | 1.25 | 4527.4 | .211 |
| Group (CG) x time | 0.03 | 0.09 | 0.09 | 2227.9 | .744 |  | -0.06 | 0.33 | -0.19 | 1597.8 | .853 |

*Significant *p*-value

^a^Subscore of the PPCI-r: Paediatric Pain Coping Inventory-revised

^b^PPDI: Pediatric Pain Disability Index

^c^API: Abdominal Pain Index (Secondary Outcome)

^d^A-PKQ: Abdominal Pain Knowledge Questionnaire (Child Version, ≥ 8 years)

## Supplementary Material S4

**Table S4**

*Explorative Per-protocol analyses. MLMs outcomes including predictors sex and age for T_1_-T_3_ measurements of all parent outcomes.*

|  | **Coefficient** | **SE** | ***t*** | **df_error_** | ***p*** |  | **Coefficient** | **SE** | ***t*** | **df_error_** | ***p*** |  |
| --- | --- | --- | --- | --- | --- | --- | --- | --- | --- | --- | --- | --- |
| **Abdominal Pain Knowledge^a^** | | | | | | **Discouragement^b^** | | | | | | |
| Intercept | 6.58 | 1.18 | 5.60 | 13298.2 | <.001 |  | 10.88 | 2.15 | 5.06 | 18259.9 | <.001 |  |
| Group (CG) | -0.84 | 0.44 | -1.89 | 85213.5 | .059 |  | 0.90 | 0.78 | 1.15 | 73677.7 | .250 |  |
| Time | 0.21 | 0.14 | 1.53 | 17546.3 | .126 |  | 0.17 | 0.22 | 0.79 | 13481.8 | .433 |  |
| Sex (female) | 0.60 | 0.46 | 1.31 | 2205.2 | .191 |  | 0.68 | 0.81 | 0.84 | 2582.9 | .400 |  |
| Age | 0.02 | 0.02 | 0.71 | 36441.6 | .476 |  | 0.02 | 0.05 | 0.45 | 62594.7 | .656 |  |
| Group (CG) x time | 0.27 | 0.19 | 1.43 | 19328.5 | .153 |  | 0.08 | 0.30 | 0.28 | 11993.2 | .781 |  |
| **Solicitousness^b^** | | | | | | **Distraction^b^** | | | | | | |
| Intercept | 22.79 | 2.16 | 10.54 | 17045.7 | <.001 |  | 15.85 | 1.41 | 11.23 | 20160.0 | <.001 |  |
| Group (CG) | -0.37 | 0.78 | -0.47 | 89460.9 | .637 |  | -1.00 | 0.55 | -1.84 | 126700.0 | .066 |  |
| Time | -1.29 | 0.22 | -5.99 | 17497.1 | **<.001*** |  | 0.26 | 0.17 | 1.55 | 35830.0 | .122 |  |
| Sex (female) | -0.43 | 0.84 | -0.52 | 1732.4 | .606 |  | 0.43 | 0.55 | 0.78 | 3272.0 | .434 |  |
| Age | 0.03 | 0.05 | 0.64 | 56978.6 | .521 |  | -0.08 | 0.03 | -2.55 | 50330.0 | **.011*** |  |
| Group (CG) x time | -0.14 | 0.30 | -0.48 | 12772.7 | .632 |  | 0.29 | 0.23 | 1.24 | 27710.0 | .214 |  |

*Significant *p*-value

^a^A-PKQ: Abdominal Pain Knowledge Questionnaire

^b^ISEV-E: Inventar zum schmerzbezogenen Elternverhalten (Pain-related Parent Behavior Inventory)
